# Use of Spectroscopic Techniques for a Rapid and Non-Destructive Monitoring of Thermal Treatments and Storage Time of Sous-Vide Cooked Cod Fillets

**DOI:** 10.3390/s20082410

**Published:** 2020-04-23

**Authors:** Abdo Hassoun, Janna Cropotova, Turid Rustad, Karsten Heia, Stein-Kato Lindberg, Heidi Nilsen

**Affiliations:** 1 Nofima, Norwegian Institute of Food, Fisheries, and Aquaculture Research, Muninbakken 9-13, 9291 Tromsø, Norway; karsten.heia@nofima.no (K.H.); stein-kato.lindberg@nofima.no (S.-K.L.); heidi.nilsen@nofima.no (H.N.); 2 Department of Biotechnology and Food Science, Norwegian University of Science and Technology, 7941 Trondheim, Norway; janna.cropotova@ntnu.no (J.C.); turid.rustad@ntnu.no (T.R.)

**Keywords:** fish, fluorescence, cooking, classification, spectroscopy, storage, prediction

## Abstract

In this work, the potential of spectroscopic techniques was studied to investigate heat-induced changes occurring during the application of thermal treatments on cod (*Gadus morhua* L.) fillets. Vacuum-packed samples were thermally treated in a water bath at 50, 60, 70 and 80 °C for 5 and 10 min, and further stored for one, four, and eight days at 4 ± 1 °C before analysis. Several traditional (including cooking loss, drip loss, texture, protein solubility, protein oxidation, and color) and spectroscopic (fluorescence and diffuse reflectance hyperspectral imaging) measurements were conducted on the same samples. The results showed a decrease in fluorescence intensity with increasing cooking temperature and storage time, while the impact of cooking time was only noticeable at low temperatures. Diffuse reflectance data exhibited a decrease in absorbance, possibly as a result of protein denaturation and increased scattering at higher cooking temperatures. Both fluorescence and diffuse reflectance data were highly correlated with color parameters, whereas moderate correlations were observed with most other traditional parameters. Support vector machine models performed better than partial least square ones for both classification of cod samples cooked at different temperatures and in prediction of the cooking temperature. The best classification result was obtained on fluorescence data, achieving an accuracy of 92.5%, while the prediction models resulted in a root mean square error of prediction of cooking temperature lower than 5 °C. Overall, the classification and prediction models showed good results, indicating that spectroscopic techniques, especially fluorescence hyperspectral imaging, have a high potential for monitoring thermal treatments in cod fillets.

## 1. Introduction

The application of thermal treatments to food products has been widely used since ancient times with the purpose of destroying pathogens and giving the food desired organoleptic properties as well as increased digestibility. Nowadays, most foods, especially muscle foods (fish, meat, etc.), should be subjected to different types of thermal treatments during processing or preparation prior to consumption. A common practice in the food industry is to overheat food in order to ensure the safety of the processed products. However, the application of more heat than required causes an unnecessarily high energy consumption resulting in a lower profitability in the seafood industry [1]. Moreover, as fish and other seafood products are highly sensitive to thermal treatments, the application of severe heat can have negative consequences on sensory attributes, such as color, texture, etc., of the processed products. For example, some studies indicated that high temperatures can lead to protein and lipid oxidation as well as significant textural and structural modifications in seafood products [2,3,4,5]. Nutritional quality parameters (e.g., digestibility) are also affected by high thermal loads, for example, as in the case of heating fish at 200 °C for an extended period of time [6,7].

Thus, applying the appropriate amount of heating, just enough to kill pathogenic microorganisms, without affecting sensory and nutritional quality of the treated products is a challenging task. Optimization of the thermal processing requires both minimal heat processing in order to avoid undesired effects of high thermal load, and efficient analytical methods that allow for a rapid detection of heat-induced changes and eventual corrective actions, if necessary [8]. Several studies have investigated different minimal heat processing methods, and the results have varied both according to the raw material quality and the applied technique, among others [9,10,11,12]. Traditionally, heat-induced changes and quality of fish and other seafoods are evaluated using sensory, microbiological, and physio-chemical analysis, such as oxidation products, color, and texture [5,13,14]. For example, the texture is a key quality parameter of fish and other seafood, and has been widely evaluated using sensory and instrumental methods [5,15,16]. However, it is well known that measuring the texture of fish is a challenging task as texture parameters, such as hardness, change along the fish as a function of its thickness [17]. Besides the specific limitations associated with each traditional method (e.g., change in thickness when measuring the texture), a more general challenge with most of these methods is that they are destructive, thus cannot be used to evaluate all products, and only a few samples are analyzed offline. On the contrary, spectroscopic techniques can be used to scan samples at line, online, or inline of production. With at line scanning, the sample is withdrawn and analyzed in the immediate environment of the industrial equipment, while in the case of online measurements, a portion of the production is analyzed via a by-pass system, then it returns to the system. The ideal solution is the inline scanning where a sensor can be placed in a process stream of flowing material, thus all the production volume is analyzed. 

Several studies have indicated that spectral changes induced by application of heat can be used to monitor thermal treatments in fish and other seafoods. Thus, the development of noncontact, non-destructive, and real-time measurement sensors based on spectroscopy can be a valuable tool in the modern food industry [8]. Previous studies have investigated the potential of visible and near infrared spectroscopy [1,8,11,12], Raman spectroscopy [18,19], and nuclear magnetic resonance (NMR) [4,20] for monitoring changes in seafood induced by the application of thermal treatments. However, only a few studies in literature have reported on the use of fluorescence spectroscopy for monitoring thermal treatments in fish and other seafood products. In the studies that have recently investigated the potential of fluorescence spectroscopy, the technique was mainly used as a complementary tool to support other results obtained from traditional measurements [7,21]. The combination of fluorescence with hyperspectral imaging makes it a promising tool for various applications in the food industry. Taking this into account, the main objective of this study is to evaluate the feasibility of using hyperspectral imaging in fluorescence mode to investigate changes in cod samples induced by heat and following chilled storage and to compare the results with diffuse reflectance spectroscopy and a selection of traditional analysis. The samples were evaluated packed while moving on a conveyor belt in order to mimic industrial processes.

## 2. Materials and Methods

### 2.1. Samples Preparation and Cooking Treatment

A Norwegian Arctic cod, labelled as “Skrei”, was obtained from Vengsøya near Tromsø (in Norway), on the day of catch. The fish was gutted, iced, and transported to Nofima (Tromsø, Norway) the same day. The next day, the fish was filleted, and only the loin parts were used for the experiments. Each fish was cut into two fillets, and two loin-pieces (samples) were obtained from each fillet. One hundred and twenty samples were obtained from 3 batches of fish (Table 1). Twenty-four samples were analyzed on day 1, 4, and 8 without cooking (control group), with half of the samples being vacuum-packed and the other half being air packed. The rest of the fish (96 samples) were divided into 8 groups, each group of 12 samples. The samples were cooked at different temperatures (50, 60, 70 or 80 °C) for 5 or 10 min. Before cooking, the samples were vacuum-packed and then cooked in a water bath preheated at the desired temperature. After cooking, the samples were cooled in iced water for 1 minute and analyzed on day 1 and stored at 4 ± 1 °C for further analysis performed on days 4 and 8. Four samples from each group and for each storage day were analyzed.

### 2.2. Traditional and Spectroscopic Measurements

Cooking loss was determined gravimetrically as the difference in weight of samples before and after the heat treatment (twelve replicates for each cooking temperature/time). The sous-vide cooked fish samples were removed from the vacuum bags and wiped gently with tissue paper to remove excess surface moisture before performing the measurements. The liquid remaining in the vacuum bag after taking out the fish was weighed and the drip loss was calculated as the percentage of fish weight loss after removing the liquid [22].

Texture profile analysis (TPA) was performed using the TA.HD plus texture analyzer (Stable Micro System) at room temperature. The test conditions were the following: two consecutive cycles of 80% compression using a flat-ended cylindrical plunger (12 mm diameter), pre-test speed: 5 mm/s, test speed: 1 mm/s, post-test speed: 10 mm/s, target mode: strain, trigger force: 5 g. The following TPA parameters were obtained from force–time curves: hardness, cohesiveness, springiness, chewiness, and resistance [16]. 

The solubility of sarcoplasmic (water-soluble) and myofibrillar (salt-soluble) proteins was determined according to the method described by Hultmann and Rustad (2002) [23]. Fish samples were ground with a benchtop mixer Bosch (750 W, Germany) and 4 g were taken for further homogenization for 20 s in 80 mL phosphate buffer (0.05 M phosphate, pH 7.0) using an Ultra Turrax (samples were kept on ice 0 + 1 °C) followed by centrifugation at 8000 g for 20 min). The resulting volume was made up to 100 mL with phosphate buffer (water-soluble fraction). The remaining precipitate was further homogenized for 10 s in phosphate buffer with KCl (0.05 M phosphate, 0.6 M KCl, 0.5% tritonX-405, pH 7.0), and centrifuged as described above. The supernatant was adjusted to 100 mL with KCl-phosphate buffer (salt-soluble fraction). The amount of proteins in the extracts of water- and salt-soluble proteins (in % wet weight fish) was determined with BioRad protein assay using gamma globulin as a standard. The analyses were run in triplicate and the mean value ± SD was calculated.

Protein carbonyl groups in sarcoplasmic and myofibrillar proteins were detected by DNPH-based Enzyme-Linked Immunosorbent Assay (ELISA) performed in a 96-well polystyrene plate as described by Cropotova and Rustad (2019) [22]. This is a rapid and highly sensitive plate-based assay technique developed by Buss and co-workers [24] based on derivatization of carbonyl groups with dinitrophenylhydrazine (DNPH) and probing of protein-bound dinitrophenyl (DNP) with an anti-DNP antibody. Carbonyl groups were determined in water- and salt-soluble proteins in quadruplicate by using the indirect ELISA kit STA-310 OxiSelectTM (Cell Biolabs Inc., San Diego, CA, USA), and the average value with standard deviation were calculated. The results were expressed in nmol carbonyls/mg protein.

The color parameters of the sous-vide cooked cod samples were determined instrumentally using a Minolta Chroma meter CR-400 (Konica-Minolta, Osaka, Japan). Before performing the measurements, the instrument was calibrated with the instrument standard white plate. Measurements of color parameters were conducted on preselected locations of each cod sample at room temperature. The data were recorded in color coordinates of L* (lightness, black = 0, white = 100), a* (redness > 0, greenness < 0), and b* (yellowness, b* > 0, blue < 0) according to the Commission Internationale de l’Éclairage (CIE) Lab scale. Color parameters were read three times on each sample and the average with standard deviation was calculated.

Fluorescence measurements were carried out using a pushbroom hyperspectral camera (VNIR-1024, Norsk Elektro Optikk, Skedsmokorset, Norway) operating in the spectral range from 410–990 nm with a spatial resolution of 0.28 mm across-track and 0.48 mm along track and a spectral resolution of 2.7 nm. A focused LED UV line light was used for the excitation with a center wavelength of 365 nm (Metaphase UL-LL409-UV365-24) and the emission was recorded at wavelengths higher than 400 nm. The camera was fitted with a lens focused at 1000 mm, mounted 1020 mm above the conveyor belt carrying the samples to be imaged. Three-dimensional hyperspectral image cubes, built line by line, were obtained by running the conveyor belt at a speed of 1 cm/s. The fluorescence data were corrected for height variations of the samples by dividing fluorescence intensity at each wavelength by the peak value of each spectrum. 

Diffuse reflectance measurements were performed using a VNIR-640 imaging camera (Norsk Elektro Optikk, Skedsmokorset, Norway) with a spatial resolution of about 0.5 × 0.5 mm and a spectral resolution of approximately 10 nm, within the spectral range of 430–1000 nm. The camera was mounted 1020 mm above a conveyor belt carrying the samples at a speed of 40 cm/s. The samples were illuminated with a custom-made light source, which consists of 14 halogen bulbs (50 W) mounted inside a box made of 10 mm thick high-density polytetrafluoroethylene (PTFE, also known as Teflon) plates [25]. 

### 2.3. Data Analysis

Multiple regression analysis was performed to determine the relationships between heat-induced changes and storage and cooking conditions. Analysis of variance (ANOVA) and Tukey’s Honestly Significant Difference (HSD) post-hoc tests were performed in the R software, and were used to test differences between sample means, which were considered significant at *p* < 0.05. Univariate correlations among the spectroscopic data and traditional parameters were evaluated by the Pearson coefficient. Principal Components Analysis (PCA) was applied on fluorescence data to investigate the ability of these measurements to separate between the samples as a function of cooking and storage conditions. 

Partial Least Square Regression (PLSR) and Support Vector Machine Regression (SVMR) models were used to predict cooking conditions and storage times from the spectroscopic data [26,27,28]. The discriminant ability of each spectral dataset was determined by applying Partial Least Square Discriminant Analysis (PLS-DA) and Support Vector Machine Classification (SVMC) models [28,29]. All the classification and the prediction models developed in this study were validated using a cross-validation procedure. The prediction and classification models were performed by using the PLS-Toolbox v.8.5 (Eigenvector Research) for MATLAB R2018a. The regions of interest were selected manually at the image center for each sample and the average spectral data were generated from the images. The extraction of data was performed in IDL 8.6 (L3Harris Technologies, Inc.).

## 3. Results and Discussion

**Figure 1:**
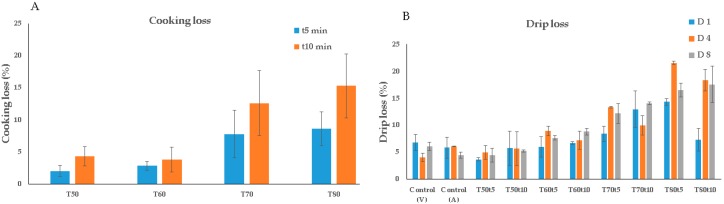
Cooking loss (**A**) and drip loss (**B**) obtained on the cod samples as a function of cooking temperature; T, cooking time; t, and storage days; D (V; vacuum-packed samples, A; air-packed samples).

**Cooking Loss**: High cooking loss has a negative impact on sensory perception of fish and other seafood. Our results showed that the cooking loss generally increased (R = 0.88, *p* < 0.05) both with increasing cooking temperature and cooking time (Figure 1A). The increase in cooking loss with increasing thermal load was previously reported in several studies and explained by protein denaturation and loss of water holding capacity [5,9,30]. However, the cooking loss levels in our study seem to be higher than those reported in literature [5,9,14,30]. For example, in the present study, the mean cooking loss value of cod samples cooked at 80 °C for 10 min exceeded 15%. This might be because the fish used in our study was still in pre-rigor, or at least in the early stage of rigor mortis (< 24 h post-slaughter) while the fish used in the other studies were in rigor state when the samples were heat-treated [10]. Regardless of cooking time, the cooking loss was significantly higher for the cod fillets cooked at 70 and 80 °C than for those cooked at 50 and 60 °C, while differences in cooking loss between the samples cooked at 50 and 60 °C and between those cooked at 70 and 80 °C were not significant. PCA applied to the cooking loss data showed a good separation between the samples as a function of both cooking temperature (according to the PC1) and cooking time (according to the PC2) (Supplementary data S1).

**Drip Loss**: In the present study, drip loss of untreated cod samples was significantly lower (*p* < 0.05) compared to the drip loss of samples subjected to heat treatment at 70 and 80 °C for both 5 and 10 min (Figure 1B). This tendency suggests that a higher thermal load more strongly affects the integrity of the myofibrillar network of cod muscle due to severe denaturation of myofibrils, thus decreasing the water holding capacity of the fish [14]. However, no significant variation was found between the cod loins cooked at 50 °C for 5 and 10 min and uncooked samples.

**Texture Parameters**: A general trend of decreased hardness was observed with storage time (Supplementary data S2), in agreement with other studies [14]. The decrease in hardness was attributed to alteration in muscle structure such as changes in myofibrillar proteins and connective tissue as well as activity of autolytic enzymes [2,31,32]. Regarding the treated samples, significantly (*p* < 0.05) higher hardness values were observed for the samples cooked at 50 °C compared to the hardness of the samples cooked at the other temperatures (60, 70, 80 °C). Significantly higher hardness values were observed for the samples cooked for five min compared to those cooked for 10 min, while significant differences were only found among the samples stored for one and four days. A similar trend was observed for the springiness and chewiness parameters. The cohesiveness, which represents the force that holds the integrity of fish structure and prevents it from gaping, was significantly lower for the samples cooked at 80 °C. The resilience was significantly affected by both storage time and cooking conditions (temperature and time). Indeed, significantly (*p* < 0.05) higher resilience values were observed for the samples cooked at 50 °C and for the samples cooked for five minutes than the resilience values of those cooked at 70 and 80 °C and the samples cooked for 10 min, respectively. 

**Protein Solubility**: The total amount of extracted sarcoplasmic (water-soluble) and myofibrillar (salt-soluble) proteins decreased significantly (*p* < 0.05) after the sous-vide cooking of cod loins compared to untreated fish samples (Supplementary data S3). This reduction indicated heat denaturation and aggregation of these proteins. However, the solubility was neither affected by cooking temperature, nor duration of cooking, since no significant difference between the samples cooked at 50, 60, 70 and 80 °C for 5 and 10 min, was found. A significant correlation (R = 0.65, *p* = 0.05) was observed between solubility of myofibrillar proteins and drip loss in sous-vide cooked cod samples. Considering that the myofibrillar network is generally responsible for holding water inside the fish muscle [23], we suggest that the decrease in solubility of salt-soluble proteins in the present study contributed to increased drip loss due to thermal denaturation and aggregation of myofibrils [14].

**Figure 2:**
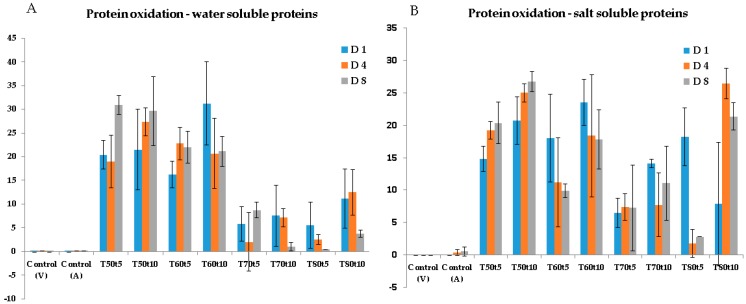
Total carbonyls in sarcoplasmic (**A**) and myofibrillar proteins (**B**), obtained on the cod samples as a function of cooking temperature; T, cooking time; t, and storage days; D (V; vacuum-packed samples, A; air-packed samples).

**Protein Oxidation**: Oxidation is one of the most important quality deterioration processes in muscle foods. Lipid oxidation has been widely investigated, while less attention has been paid to protein oxidation and other oxidative damages. Although oxidation products can be generated from the other compounds in cod (proteins, carbohydrates, etc.), protein oxidation is most likely the dominant pathway in this lean fish. The total carbonyl content increased significantly (*p* < 0.05) both in sarcoplasmic and myofibrillar proteins of sous-vide cooked cod samples compared to uncooked samples (Figure 2). This increase can be explained by the interaction of proteins with various pro-oxidants (free iron, myoglobin, etc.) released from heat-disrupted cells during heat treatment, as well as lipid oxidation products formed during storage of the fish. According to earlier investigations [33], thermal load disturbed the integrity of the fish muscle structure through cell rupture, leading to further oxidation reactions between unsaturated fatty acids, heme pigments (myoglobin) and other pro-oxidants free iron, etc.). The oxidized lipids reacted further with proteins, resulting in their carbonylation. However, cod samples cooked at 50 and 60 °C had significantly higher (*p* < 0.05) carbonyl content in sarcoplasmic proteins compared to fish samples cooked at 70 and 80 °C. This tendency can be explained by thermal inactivation of prooxidative endogenous enzymes that increase oxidation of fish [33]. 

**Figure 3:**
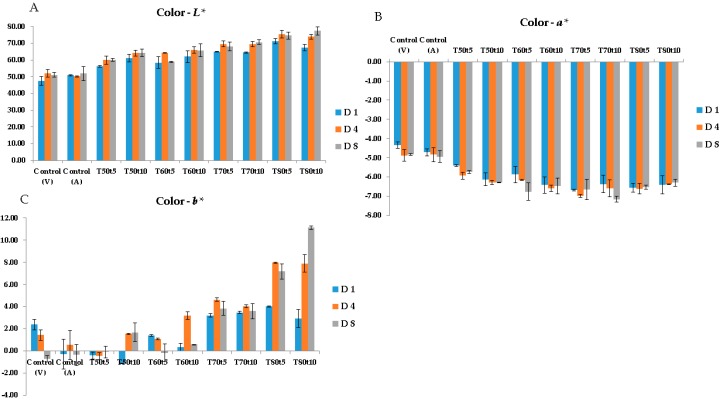
Color parameters: lightness (**A**), redness (**B**), and yellowness (**C**), obtained on the cod samples as a function of cooking temperature; T, cooking time; t, and storage days; D (V; vacuum-packed samples, A; air-packed samples).

**Changes in Color**: The lightness (L*-value) of the sous-vide cooked cod increased significantly (*p* < 0.05) along with a gradual decrease in redness (a*-values) during the storage period (Figure 3A,B) compared to the color parameters of untreated cod samples. These results are in agreement with the previous study of Cropotova and others [14] performed on sous-vide cooked Atlantic mackerel. A possible explanation for the increased lightness and consecutively decreased redness of cod flesh may be denaturation and aggregation of heme-proteins [14]. It should be mentioned that modifications of optical characteristics of the muscle tissue, induced by the application of high temperatures, as will be shown later, can also contribute to the observed changes in color. A large variation in yellowness (b*-value) was observed during storage of sous-vide cooked cod samples (Figure 3C). The variation in yellowness could probably be due to accumulation of lipid–protein oxidation products, including the ones formed from the interaction between aldehyde groups generated during oxidation of lipids with free amino groups of phospholipids and proteins [33]. Most probably, thermal treatment caused rupture of the cell membranes of adipocytes, thereby liberating lipid oxidation products that further came in contact with proteins leading to protein oxidation and change in color of the cod flesh. This hypothesis is supported by a significant correlation between b*-value and amount of protein carbonyls generated in both sarcoplasmic (R = −80, *p* < 0.05) and myofibrillar proteins (R = −0.69, *p* < 0.05). 

A PCA was carried out on the mean values of all traditional measurements, including protein oxidation, protein solubility, texture, color, and drip loss data (Supplementary data S4). The first two principal components accounted for 62.29% of the total variance (41.83% for the PC1 and 20.46% for the PC2). The PCA score plot showed a clear separation of the control group from the cooked cod samples. Moreover, the cod samples cooked at low temperatures (50 and 60 °C) were well separated from those cooked at high temperatures (70 and 80 °C). The loading of the PCA revealed that the most influencing parameter on the control group was the proteins solubility, positively correlated with the PC1. The low-temperature cooked samples were mostly affected by protein oxidation and cohesiveness, positively correlated with the PC2, while the high-temperature cooked samples were mainly influenced by drip loss and L* color, negatively correlated with the PC2. 

**Fluorescence Spectra**: Compared to other spectroscopic techniques, the fluorescence spectroscopy is very sensitive to changes in the local molecular environment of fluorophores, such as changes in pH, temperature, polarity, and color [34,35,36]. Fish can be considered as a multifluorophoric matrix due to their content of several fluorophores, such as aromatic amino acids, nicotinamide adenine dinucleotide (NADH), riboflavin, oxidation products, collagen, among others.

**Figure 4:**
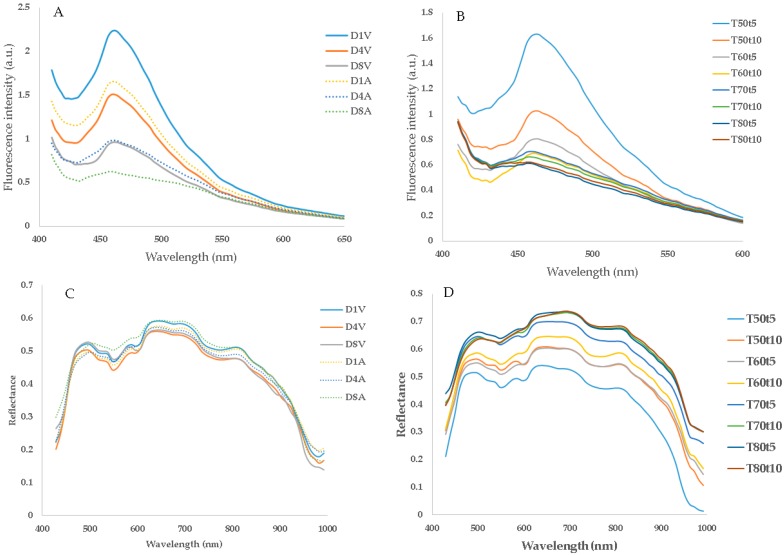
Mean fluorescence (**A**,**B**) and diffuse reflectance (**C**,**D**) spectra of the control (A and C) and the heat treated samples (B and D), obtained as a function of storage days (D), packaging types (V; vacuum-packed samples, A; air-packed samples), and cooking conditions; cooking temperature; T, cooking time; t.

The Figure 4A shows that both the storage time and packaging type have important impact on fluorescence spectra. Although no shift in the fluorescence maximum peak position was seen, fluorescence emission around 460 nm seemed to decrease with increasing storage time in both packaging types, and the fluorescence emission of vacuum-packed samples is higher than that of air-packed samples. Previous studies also reported that storage time and packaging type influence fluorescence spectra [37,38,39]. Figure 4B displays changes in fluorescence spectra as a function of cooking temperature and cooking time. Other studies have shown that heating temperature and time have an important impact on fluorescence spectra [7,21,40]. The results show that the impact of time (5 and 10 min) is clearer at low temperatures (50 and 60 °C), than at high temperatures (70 and 80 °C) probably due to a complete denaturation of proteins after cooking at higher temperature. Several studies have shown that the denaturation of proteins in fish and other seafood increases with increasing cooking temperature and time [14,41]. The impact of cooking conditions (temperature and time) was studied along with the subsequent storage time, using classical multiple linear regressions. The mean fluorescence emission at 461 nm was set as the dependent variable (input Y), while cooking temperature, cooking time, and storage time were set as independent parameters (inputs X1, X2, X3), and the following Equation (1) (Supplementary data S5) was derived:

(1) $$F=3.64-0.026T-0.048d\;(R^{2}=0.77)$$

where F = fluorescence intensity at the maximum emission (arbitrary unit), T = cooking temperature (°C), d = duration of chilled storage (day). 

The results show that increasing both cooking temperature and storage time induced a decrease in fluorescence emission, while the contribution of cooking time was not significant. This decrease can be due to an increase in protein denaturation and a decrease in collagen content, since fluorescence around this emission wavelength (around 460 nm) can be attributed to collagen [42,43]. Recently, Cropotova and coresearchers noticed a significant decrease in collagen content of sous-vide cooked mackerel during chilled storage [2]. Besides the changes in fluorescence intensity, a blue shift in the maximum peak position of about 4 nm and 6 nm was observed for the mean spectra of samples cooked at 70 and 80 °C, respectively, compared to the control and low-temperature heated samples, which might indicate a less polar environment for the samples heated at high temperatures. 

**Visible Near-Infrared Diffuse Reflectance Spectra**: Diffuse reflectance spectra have several advantages, including (among others), the possibility of scanning samples online (on a conveyor belt) when only one surface is available, and illuminating the sample ideally without shadow on the surface or the background [44]. The mean reflectance spectra of cod samples packed with or without vacuum and stored for different times are shown in Figure 4C. The spectra have a similar general trend with small differences in spectral shape and reflectance values. The main peaks in the visible and near infrared parts of the spectra have been attributed to water and different oxidation states of haemoglobin [25,45,46]. Figure 4D shows the mean spectra of cod samples cooked at different temperatures and durations. It can be observed that the samples cooked at the highest temperature have the lowest absorbance value (highest reflectance) and vice versa. These results agree with those reported in previous studies and are explained by changes in optical characteristics, such as absorption and scattering properties, caused mainly by protein denaturation, loss in water binding, and other structural changes [8,11,12]. In more detail, the interaction of light with biological tissue is the result of a complex interplay between light absorption and light scattering, representing the possibility of a photon being respectively absorbed and scattered inside the sample. The scattering is associated with the structural and physical properties of the sample, while the absorption is mainly related to the chemical composition and concentration of the various chemical components that absorb light at different wavelengths in different manners. Upon heating, systematic changes in the spectra can be seen, with more scattering and less intense absorption, due to less absorbance of water, proteins, and other organic compounds. More specifically, an increase in cooking temperature causes an increase in scattering particles, as a result of protein denaturation, which in turn decreases absorption from functional groups in water and proteins. These changes and alteration in optical properties, caused by the high temperatures, impacts several physical properties of fish muscle, such as changes in color and texture.

A classical multiple linear regression was carried out to explain the impact of cooking conditions and storage time on diffuse reflectance spectra, and the following model (2) (Supplementary data S5) was obtained:

(2) $$\mathrm{DR}=1.096-0.0064T-0.0078d\;(R^{2}=0.84)$$

where DR = diffuse reflectance (mean value), T = cooking temperature (°C), d = duration of chilled storage (day). 

Again, for the fluorescence spectra, the diffuse reflectance spectra were significantly affected by cooking temperature and storage time, while the impact of cooking time was not significant. Indeed, the diffuse reflectance spectra show a small difference between samples cooked for 5 and 10 min when cooking temperature was high. As only two cooking time durations (5 and 10 min) were considered in this study, a wider range with a longer time should be tested in future work. The changes induced in the spectra of samples treated thermally were attributed to modifications in the environment of protein structure (especially the secondary structure, due to the denaturation of proteins) and to changes in the state of water [45].

**Correlation Between Traditional Measurements and Spectroscopic Results**: Pearson’s correlations coefficients among the traditional measurements and the spectroscopic data were calculated. The correlations between the measurements were investigated for both the chilled stored samples (control group) and the treated samples as a function of storage time and cooking conditions (cooking temperature and cooking time). 

*Correlation Between Fluorescence Data and Traditional Measurements*: The correlations between fluorescence data and reference measurements of the control samples were first investigated. A moderate-to-strong correlation was observed between most reference measurements and fluorescence emission around the wavelengths 450 nm and 500 nm. For example, high correlations were noticed between fluorescence data around the maximum emission and protein solubility (R = −0.94), a* color parameter (R = 83), springiness (R = 82), and resilience (R = 81), whereas lower correlations were observed with carbonyls in salt-soluble proteins (R = −75) and the other texture and color parameters (with R values varying between 0.65 and 0.79). Other studies have also reported correlations between fluorescence measurements and references analysis performed on fish during chilled storage [47], frozen storage [48], or storage under different conditions [31,38]. Subsequently, in our study, the correlation was developed in order to investigate relationship between the fluorescence and reference measurements performed on the heat-treated samples. The results revealed different patterns with varying efficiency according to the fluorescence emission wavelength used for the correlation and the studied reference parameter. For example, the fluorescence intensity around the maximum emission (461 nm) showed low correlation values with carbonyls in water-soluble proteins (R = 0.64), hardness (R = 0.57), and chewiness (R = 0.60), while a stronger correlation was observed with resilience (R = 0.80). However, color parameters, especially the lightness parameter (R = −0.89), as well as the drip loss (R = −0.79), seemed to correlate well with the fluorescence data. The results suggest that the fluorescence spectroscopy can be used to assess changes in fish quality induced by storage time or thermal treatments.

*Correlation Between Diffuse Reflectance Data and Traditional Measurements*: Relationships between the diffuse reflectance data and the reference measurements, performed on the control cod samples as a function of storage time, were first examined. The results of proteins solubility (water-soluble protein and salt-soluble protein) and protein oxidation (carbonyls in salt-soluble proteins) showed good correlations with diffuse reflectance data. On the contrary to fluorescence data, diffuse reflectance data did not show statistically significant correlations with the texture parameters. These results indicate that combining fluorescence and diffuse reflectance data can give more efficient results, as some traditional parameters seem to be related to fluorescence data while others correlate better with the diffuse reflectance measurements. Recent studies have also suggested that combinations of several spectroscopic techniques can complement each other and enhance the obtained results [49,50]. Regarding the treated samples, moderate-to-high correlations were observed between the diffuse reflectance data and some reference measurements (wavelengths around 675 nm), such as resilience (R = 0.74), carbonyls in water-soluble proteins (R = 0.76), and drip loss (R = −0.90). The color parameters, especially L* (R = −0.96) and b* (R = −0.86) displayed high correlations with the diffuse reflectance data, in agreement with previous results [25], showing that diffuse reflectance measurements are highly affected by color of the samples. 

**Figure 5:**
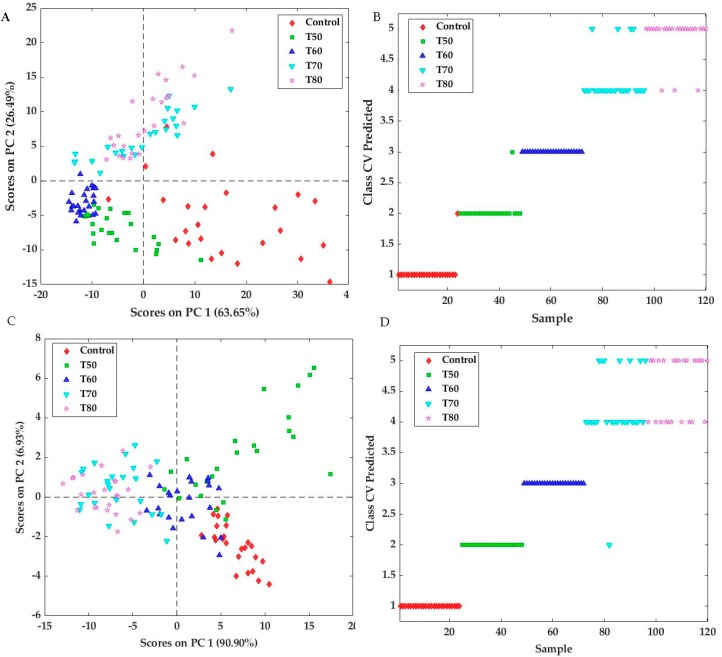
Principal Components Analysis (PCA) (**A**,**C**) and predicted cross-validated classes resulted from Support Vector Machine Classification (SVMC) analysis (**B**,**D**) applied respectively to the fluorescence (A,B) and the diffuse reflectance (C,D) data, obtained on the control and heat treated cod samples as a function of cooking temperatures (T50, T60, T70, T80).

**Multivariate Analysis and Modelling**: Univariate correlations are not always suitable and reliable for studying complex matrices like fish, which could involve possible biological variations in raw materials and undergo different handling during processing. Therefore, multivariate analysis by means of PCA as well as classification and prediction models were applied. In the first step, PCA was applied to the fluorescence and diffuse reflectance datasets. The score plot of the first two principal components (PC1 and PC2) of the PCA applied to the fluorescence spectra is shown in Figure 5A. It can be noted from this plot that the raw fish samples (control group) are well separated from fish samples cooked at high temperatures (70 and 80 °C) or low temperatures (50 and 60 °C), which in turn are well separated from each other. Similar results were obtained from the PCA applied to the diffuse reflectance spectra (Figure 5C). However, some overlapping can be observed between samples of the different groups, especially between the samples cooked at 50 and 60 °C and those cooked at 70 and 80 °C, which may be due to variations in the raw material (before cooking) or possible inconsistency during application of thermal treatments. These results are in agreement with those obtained with the PCA carried out on the traditional data (Supplementary data S4). 

In the second step, the ability of fluorescence and diffuse reflectance spectra for classifying the cod samples as a function of cooking temperatures was evaluated by means of the PLS-DA and SVMC models. PLS-DA models applied to the fluorescence and diffuse reflectance spectra showed almost a similar performance with overall correct classification rates of 84.16% and 80% for the fluorescence and diffuse reflectance models, respectively (Table 2). However, the specificity and sensitivity of the discriminant models applied to fluorescence and diffuse reflectance spectra were different. Indeed, no value lower than 0.80 was obtained for the models based on the fluorescence data, whereas values of 0.76 were observed for the prediction of the class T70 (Table 2), indicating a lower performance of the PLS-DA model based on the diffuse reflectance data compared to the fluorescence results. Overall, the models based on the two spectroscopic data showed high values (> 0.8) of Area Under the Curve (AUC), indicating that these models were promising and robust [28,51]. From the cross-validated predicted classes (Supplementary data S6 and S7) it can be observed that only the samples of the control group are located above the red dotted line, indicating an excellent model for this class, while a lower performance can been seen for the groups T50 and T60, and more overlapping for the classes T70 and T80.

Better results were obtained by application of the SVMC models using a radial basis function kernel, with the best results being observed using the fluorescence spectral data with a correct classification rate of 92.5% compared to 85.83% obtained on the diffuse reflectance data (Table 3). Indeed, only nine samples were misclassified using fluorescence data, whereas using diffuse reflectance data, 18 samples were attributed to incorrect classes. The cross validation predicted classes (Figure 5B,D) showed that the samples of the control group and the samples heated at 50 and 60 °C were well attributed to the corresponding classes. However, most of the samples heated at 70 and 80 °C were misclassified with each other. The overlapping between the samples heated at 70 and 80 °C can also be visualized by the predicted probability, which can be obtained by plotting the sample number on the abscissa and the prediction probability for each class on the ordinate (Supplementary data S8 and S9). This may indicate that the spectroscopic techniques used in this study, particularly the diffuse reflectance spectroscopy, can be especially suitable for the classification of samples heated at high (70 and 80 °C) or low (50 and 60 °C) temperatures. 

**Figure 6:**
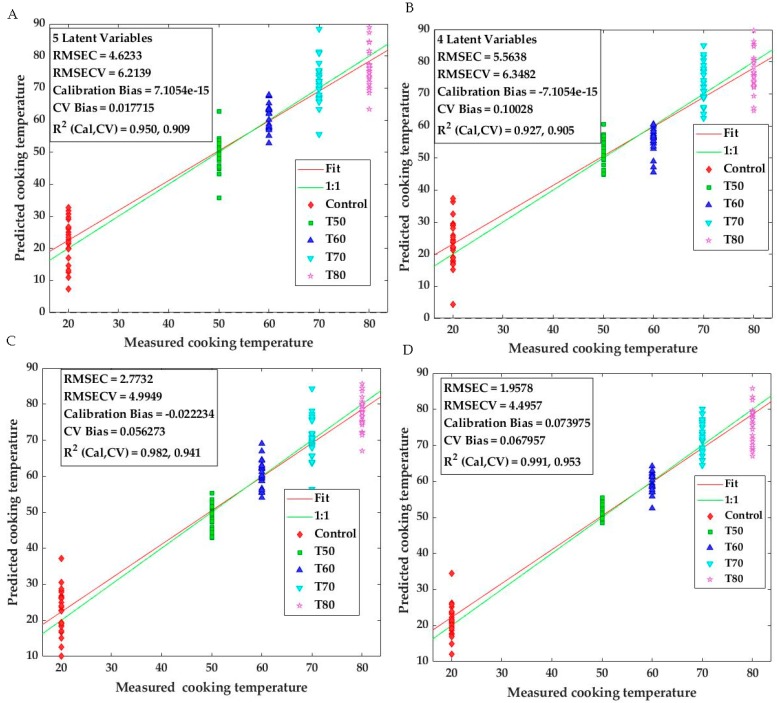
Partial Least Square Regression (PLSR) (**A**,**B**) and Support Vector Machine Regression (SVMR) (**C**,**D**) results of predicted cooking temperatures, applied respectively to the fluorescence (A and C) and the diffuse reflectance (B and D) data obtained on the control (T20) and heat treated cod samples as a function of cooking temperatures (T50, T60, T70, T80).

In the last step, PLS and SVM regressions were conducted to establish models for predicting cooking temperatures. In this case, the cooking temperature was treated as a dependent variable (Y-variable) that was predicted from the spectroscopic measurements. The models derived from fluorescence and diffuse reflectance spectra pre-treated using Extended Multiplicative Signal Correction (EMSC) and the second derivative are shown in Figure 6. The results demonstrated that the PLSR models yielded satisfactory results with R^2^ of 0.95, 0.91 and 0.93, 0.90 for calibration and validation data sets of respectively fluorescence and diffuse reflectance spectra (Figure 6A,B). Root Mean Squared Error Cross Validation (RMSECV) was 6.21 °C and 6.35 °C for the models applied to fluorescence and diffuse reflectance data, respectively. Better results were obtained by the application of SVM regression models to both datasets (Figure 6C,D). Indeed, very good fits of the models with cross-validation R^2^ of 0.94, 0.95 and RMSECV of 4.99 °C, 4.50 °C were achieved for the fluorescence and diffuse reflectance data, respectively. Similar results were reported in literature for the prediction of thermal treatments in other seafoods [1,8]. 

Less accurate results were obtained when the classification and the prediction models were applied to the spectroscopic data used to classify samples and predict cooking time and storage duration. For example, by applying the regressions models to the fluorescence data, the storage time was predicted with R^2^ of 0.82, 0.90 and RMSECV of 1.2 days, 0.89 day for the PLS and SVM regressions, respectively. Regarding the diffuse reflectance data, R^2^ of 0.77, 0.88 and RMSECV of 1.38 days, 0.99 day were obtained for respectively the PLS and SVM regressions models.

## 4. Conclusions

The findings point out that the spectroscopic techniques used in this study enable a differentiation between samples as a function of cooking temperature, whereas less accuracy can be obtained for cooking time and storage time. The support vector machine classification and prediction models used in this study seem to work better than the partial least square models. Fluorescence spectra showed a better performance than diffuse reflectance spectra for samples classification, with an accuracy rate higher than 92%, while a similar performance of the two spectroscopic techniques was observed for the prediction of cooking temperature. However, it should be noted that measurements performed by diffuse reflectance hyperspectral imaging are faster than scanning by fluorescence hyperspectral imaging. The results can be improved by taking the spatial information into consideration in future studies. Moreover, other acquisition modes, such as interactance and transmittance, can be tested in further investigations.

## Figures and Tables

**Table 1:** Summary of the experimental design.

Treatments		D1	D4	D8	Total Number
**Control**	**V**	D1TxtxV (n = 4)	D4TxtxV (n = 4)	D8TxtxV (n = 4)	n = 12
	**A**	D1TxtxA (n = 4)	D4TxtxA (n = 4)	D8TxtxA (n = 4)	n = 12
**T50**	**5 min**	D1T50t5 (n = 4)	D4T50t5 (n = 4)	D8T50t5 (n = 4)	n = 12
	**10 min**	D1T50t10 (n = 4)	D4T50t10 (n = 4)	D8T50t10 (n = 4)	n = 12
**T60**	**5 min**	D1T60t5 (n = 4)	D4T60t5 (n = 4)	D8T60t5 (n = 4)	n = 12
	**10 min**	D1T60t10 (n = 4)	D4T60t10 (n = 4)	D8T60t10 (n = 4)	n = 12
**T70**	**5 min**	D1T70t5 (n = 4)	D4T70t5 (n = 4)	D8T70t5 (n = 4)	n = 12
	**10 min**	D1T70t10 (n = 4)	D4T70t10 (n = 4)	D8T70t10 (n = 4)	n = 12
**T80**	**5 min**	D1T80t5 (n = 4)	D4T80t5 (n = 4)	D8T80t5 (n = 4)	n = 12
	**10 min**	D1T80t10 (n = 4)	D4T80t10 (n = 4)	D8T80t10 (n = 4)	n = 12

D; storage days (D1, D4, D8), T; cooking temperature (T50, T60, T70, T80), t; cooking time (5 min, 10 min), V; vacuum-packed samples, A; air-packed samples. Total number of samples = 120.

**Table 2:** Results of Partial Least Square Discriminant Analysis (PLS-DA) models applied to the fluorescence and diffuse reflectance data sets.

	Control	T50	T60	T70	T80
	**Fluorescence**				
**Sensitivity (Cal)**	0.95	0.92	0.96	0.92	0.96
**Specificity (Cal)**	1.00	0.88	0.92	0.84	0.80
**Sensitivity (CV)**	0.96	0.87	0.96	0.87	0.83
**Specificity (CV)**	1.00	0.86	0.91	0.82	0.81
**Predicted Control**	23	0	0	0	0
**Predicted T50**	0	20	1	0	0
**Predicted T60**	0	4	23	0	0
**Predicted T70**	0	0	0	18	7
**Predicted T80**	1	0	0	6	17
	**Diffuse reflectance**				
**Sensitivity (Cal)**	1.00	1.00	0.96	0.88	0.96
**Specificity (Cal)**	1.00	0.95	0.93	0.76	0.83
**Sensitivity (CV)**	1.00	1.00	0.96	0.79	0.79
**Specificity (CV)**	1.00	0.94	0.90	0.76	0.83
**Predicted Control**	24	0	0	0	0
**Predicted T50**	0	23	2	1	0
**Predicted T60**	0	1	22	0	3
**Predicted T70**	0	0	1	10	4
**Predicted T80**	0	0	0	13	17

**Table 3:** Results of support vector machines classification (SVMC) models applied to the fluorescence and diffuse reflectance data sets.

	Control	T50	T60	T70	T80
	**Fluorescence**				
**Sensitivity (Cal)**	1.00	0.96	1.00	0.91	0.87
**Specificity (Cal)**	1.00	1.00	0.99	0.97	0.98
**Sensitivity (CV)**	0.96	0.96	1.00	0.83	0.87
**Specificity (CV)**	1.00	0.99	0.99	0.97	0.96
**Predicted Control**	23	0	0	0	0
**Predicted T50**	1	23	0	0	0
**Predicted T60**	0	1	24	0	0
**Predicted T70**	0	0	0	20	3
**Predicted T80**	0	0	0	4	21
	**Diffuse reflectance**				
**Sensitivity (Cal)**	1.00	1.00	1.00	0.75	1.00
**Specificity (Cal)**	1.00	1.00	1.00	1.00	0.94
**Sensitivity (CV)**	1.00	1.00	1.00	0.67	0.58
**Specificity (CV)**	1.00	0.99	1.00	0.90	0.93
**Predicted Control**	24	0	0	0	0
**Predicted T50**	0	24	0	1	0
**Predicted T60**	0	0	24	0	10
**Predicted T70**	0	0	0	16	14
**Predicted T80**	0	0	0	7	0

## Supplementary Materials

The following are available online at https://www.mdpi.com/1424-8220/20/8/2410/s1, **Figure S1**: Biplot of the PCA applied to the cooking loss data, obtained on the cod samples as a function of cooking temperature (T50, T60, T70, T80) and cooking time (t5 min, t10 min). **Table S2**: Texture parameters obtained on the cod samples as a function of cooking temperature; T, cooking time; t, and storage days; D (V; vacuum-packed samples, A; air-packed samples). *No unit for the cohesiveness, springiness, and resilience parameters.* **Figure S3**: Water-soluble proteins (A) and salt-soluble proteins (B), obtained on the cod samples as a function of cooking temperature; T, cooking time; t, and storage days; D (V; vacuum-packed samples, A; air-packed samples). **Figure S4**: The PCA applied to the traditional data: T; temperature (T50, T60, T70, T80), V; vacuum-packed samples, A; air-packed samples. **Table S5**: Multiple linear regression statistics. **Figure S6**: Cross-validated predicted class resulted from application of PLSDA on the fluorescence data, obtained on the different cod groups: A; Control, B; T50, C; T60, D; T70, E; T80. **Figure S7**: Cross-validated predicted class resulted from application of PLSDA on the diffuse reflectance data, obtained on the different cod groups: A; Control, B; T50, C; T60, D; T70, E; T80. **Figure S8**: Prediction probability of the SVMC model applied to the fluorescence data, obtained on the different cod groups: A; Control, B; T50, C; T60, D; T70, E; T80. **Figure S9**: Prediction probability of the SVMC model applied to the diffuse reflectance data, obtained on the different cod groups: A; Control, B; T50, C; T60, D; T70, E; T80.
