# Supplementary material for: Use of Spectroscopic Techniques for a Rapid and Non-Destructive Monitoring of Thermal Treatments and Storage Time of Sous-Vide Cooked Cod Fillets
Source: Sensors (Basel). 2020 Apr 23;20(8):2410. doi: 10.3390/s20082410 (PMC7219502; doi:10.3390/s20082410)
Supplement: Supplementary file 1 [file sensors-20-02410-s001.pdf]

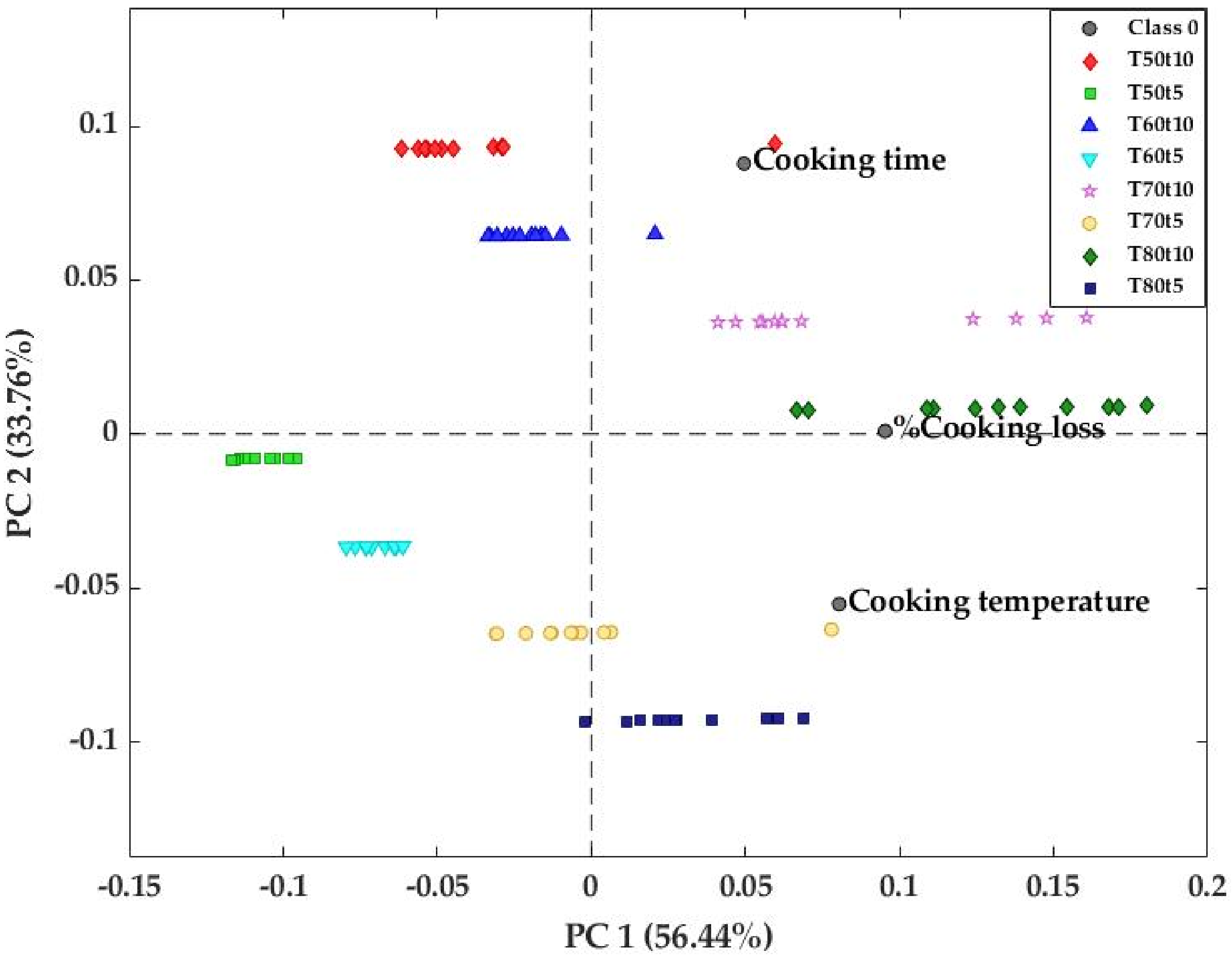

S1: Biplot of the PCA applied to the cooking loss data, obtained on the cod samples as a function of cooking temperature (T50, T60, T70, T80) and cooking time (t5 min, t10 min).

S2: Texture parameters obtained on the cod samples as a function of cooking temperature; T, cooking time; t, and storage days; D (V; vacuum-packed samples, A; air-packed samples). *No unit for the cohesiveness, springiness, and resilience parameters.*

Treatments	Storage days	Hardness (g)	Chewiness (g)	Cohesiveness	Springiness	Resilience
**V**	**1**	1172.62	347.82	0.26	0.82	0.08
**V**	**4**	952.14	238.56	0.25	0.75	0.08
**V**	**8**	717.50	220.79	0.32	0.67	0.11
**A**	**1**	559.35	162.83	0.22	0.80	0.06
**A**	**4**	571.21	191.44	0.32	0.77	0.09
**A**	**8**	650.86	183.30	0.31	0.66	0.11
**T50**	**1**	1341.33	483.58	0.36	0.81	0.11
**T50**	**4**	421.98	120.17	0.26	0.74	0.08
**T50**	**8**	741.33	251.19	0.30	0.80	0.09
**T50**	**1**	721.72	235.68	0.35	0.71	0.10
**T50**	**4**	333.42	86.26	0.31	0.67	0.09
**T50**	**8**	486.20	147.23	0.25	0.87	0.06
**T60**	**1**	619.98	206.05	0.37	0.73	0.11
**T60**	**4**	456.27	131.20	0.33	0.70	0.10
**T60**	**8**	661.69	217.49	0.30	0.87	0.07
**T60**	**1**	311.41	89.93	0.36	0.63	0.08
**T60**	**4**	424.62	102.51	0.25	0.74	0.06
**T60**	**8**	579.25	204.16	0.31	0.92	0.06
**T70**	**1**	617.08	207.59	0.36	0.75	0.08
**T70**	**4**	627.30	180.94	0.29	0.78	0.07
**T70**	**8**	486.66	163.69	0.37	0.74	0.08
**T70**	**1**	341.44	86.48	0.35	0.60	0.08
**T70**	**4**	350.79	70.12	0.29	0.58	0.07
**T70**	**8**	523.85	151.27	0.37	0.67	0.07
**T80**	**1**	541.73	150.47	0.30	0.74	0.07
**T80**	**4**	608.10	170.78	0.25	0.80	0.06
**T80**	**8**	504.86	159.69	0.29	0.84	0.05
**T80**	**1**	434.49	116.59	0.27	0.73	0.06
**T80**	**4**	369.52	80.56	0.29	0.64	0.06
**T80**	**8**	451.38	131.77	0.31	0.73	0.06

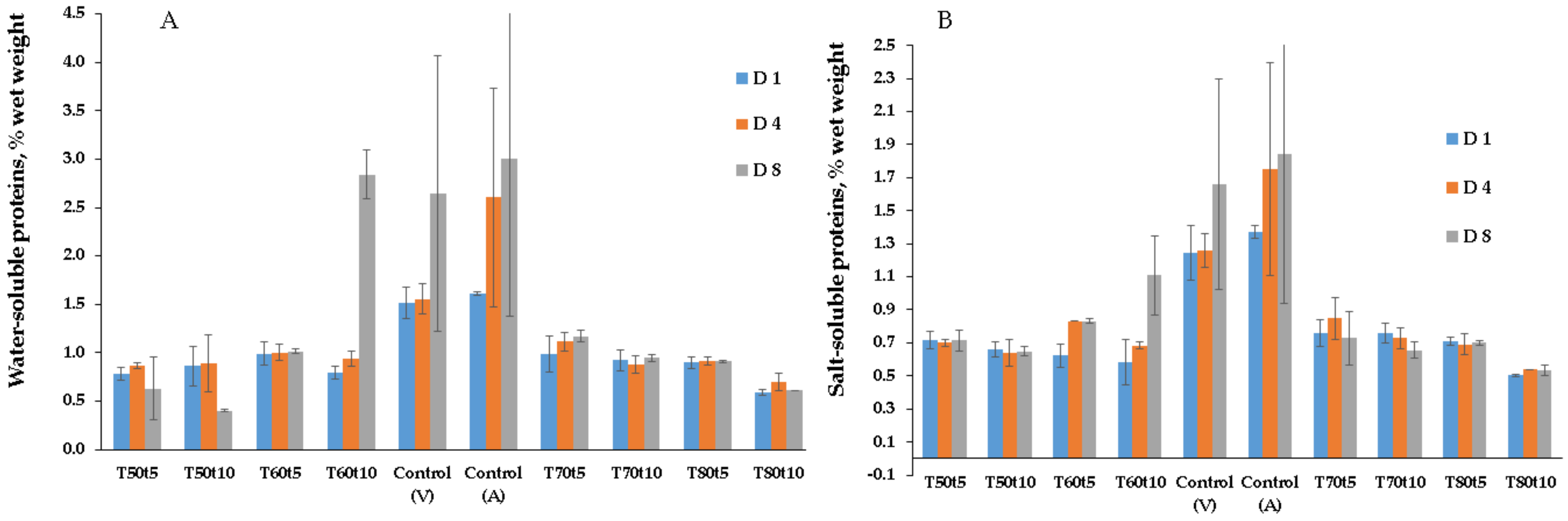

S3: Water-soluble proteins (A) and salt-soluble proteins (B), obtained on the cod samples as a function of cooking temperature; T, cooking time; t, and storage days; D (V; vacuum-packed samples, A; air-packed samples).

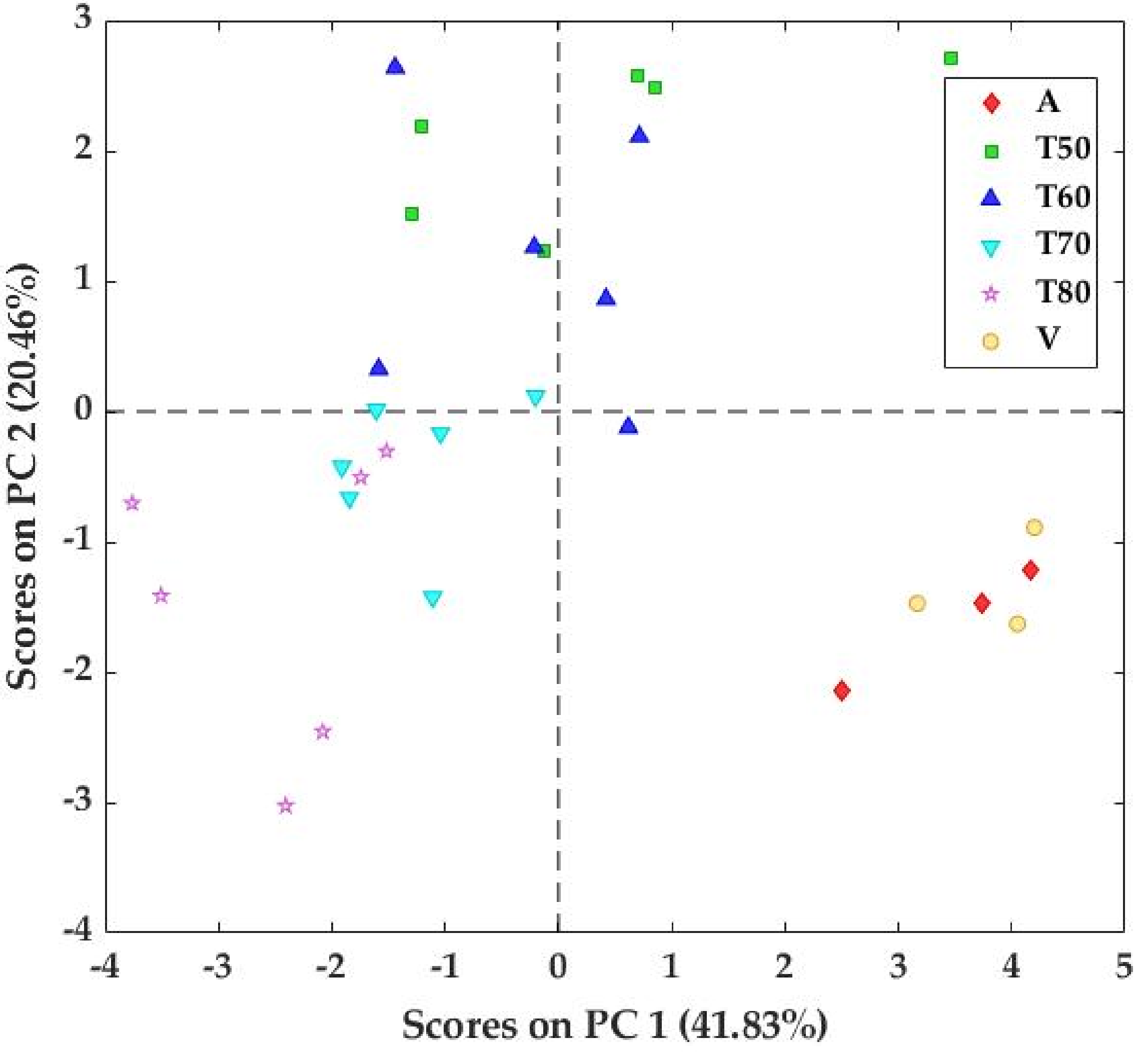

S4: The PCA applied to the traditional data: T; temperature (T50, T60, T70, T80), V; vacuum-packed samples, A; air-packed samples.

S5: Multiple linear regression statistics

A: Fluorescence data

SUMMARY OUTPUT

Regression Statistics	
Multiple R	0.894581
R Square	0.800276
Adjusted R Square	0.770317
Standard Error	0.179043
Observations	24

ANOVA

	df	*SS*	*MS*	*F*	*Significance F*
Regression	3	2.568951	0.856317	26.71272	3.38E-07
Residual	20	0.64113	0.032057		
Total	23	3.210082			

	Coefficients	*Standard Error*	*t Stat*	*P-value*	*Lower 95%*	*Upper 95%*	*Lower 95.0%*	*Upper 95.0%*
Intercept	3.635294	0.2481	14.65254	3.7E-12	3.117767	4.152821	3.117767	4.152821
T	-0.02582	0.003269	-7.89798	1.42E-07	-0.03264	-0.019	-0.03264	-0.019
t	-0.02798	0.014619	-1.91415	0.070019	-0.05848	0.002512	-0.05848	0.002512
d	-0.04785	0.012746	-3.75449	0.001248	-0.07444	-0.02127	-0.07444	-0.02127

B: Diffuse reflectance data

SUMMARY OUTPUT

Regression Statistics	
Multiple R	0.928716
R Square	0.862514
Adjusted R Square	0.841891
Standard Error	0.03322
Observations	24

ANOVA

	df	*SS*	*MS*	*F*	*Significance F*
Regression	3	0.138466	0.046155	41.82307	8.35E-09
Residual	20	0.022072	0.001104		
Total	23	0.160538			

	Coefficients	*Standard Error*	*t Stat*	*P-value*	*Lower 95%*	*Upper 95%*	*Lower 95.0%*	*Upper 95.0%*
Intercept	1.095726	0.046033	23.8029	3.8E-16	0.999702	1.191749	0.999702	1.191749
T	-0.00643	0.000607	-10.6045	1.17E-09	-0.0077	-0.00517	-0.0077	-0.00517
t	-0.00387	0.002712	-1.42838	0.168615	-0.00953	0.001784	-0.00953	0.001784
d	-0.00783	0.002365	-3.3125	0.003475	-0.01277	-0.0029	-0.01277	-0.0029

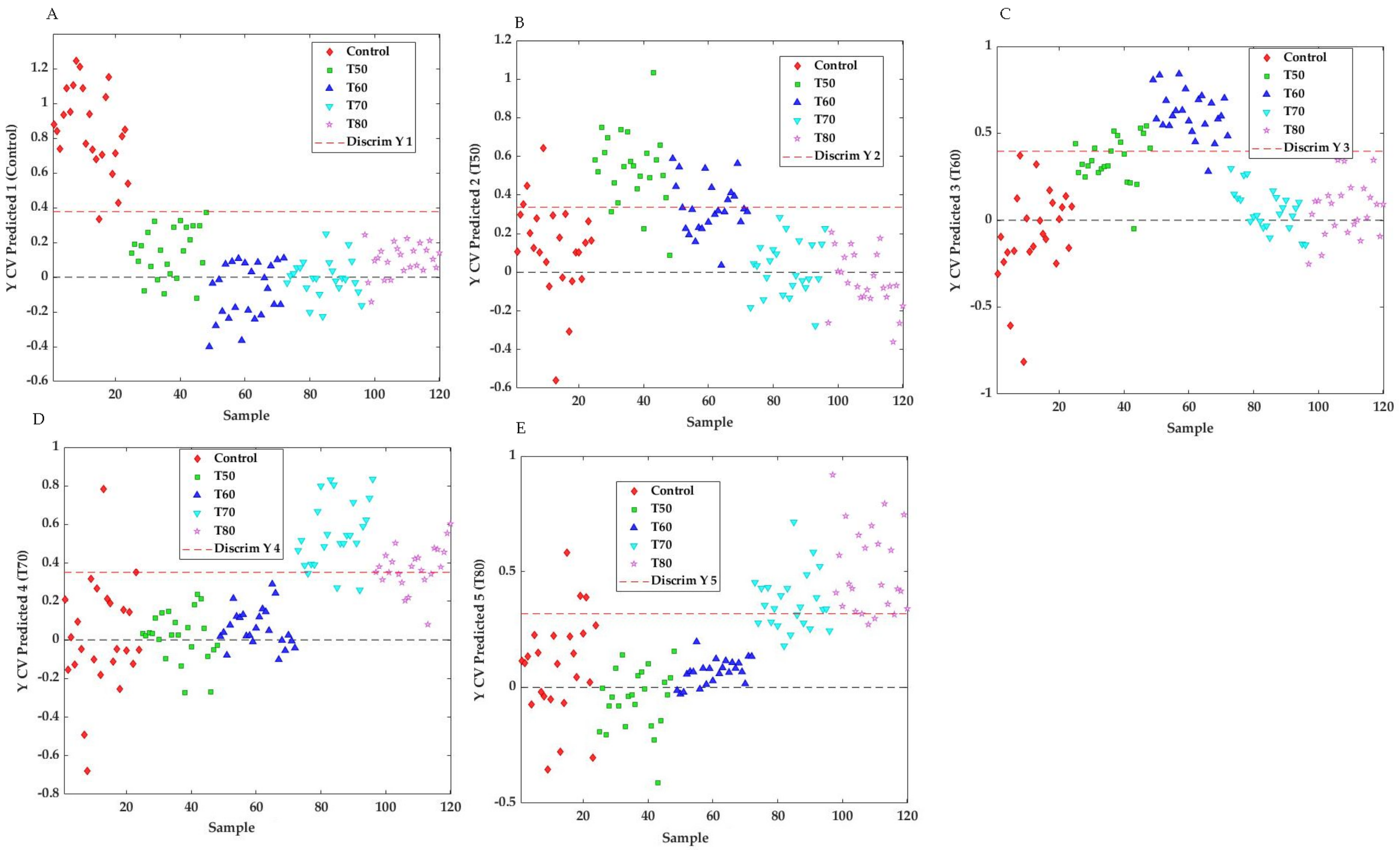

S6: Cross-validated predicted class resulted from application of PLSDA on the fluorescence data, obtained on the different cod groups: A; Control, B; T50, C; T60, D; T70, E; T80.

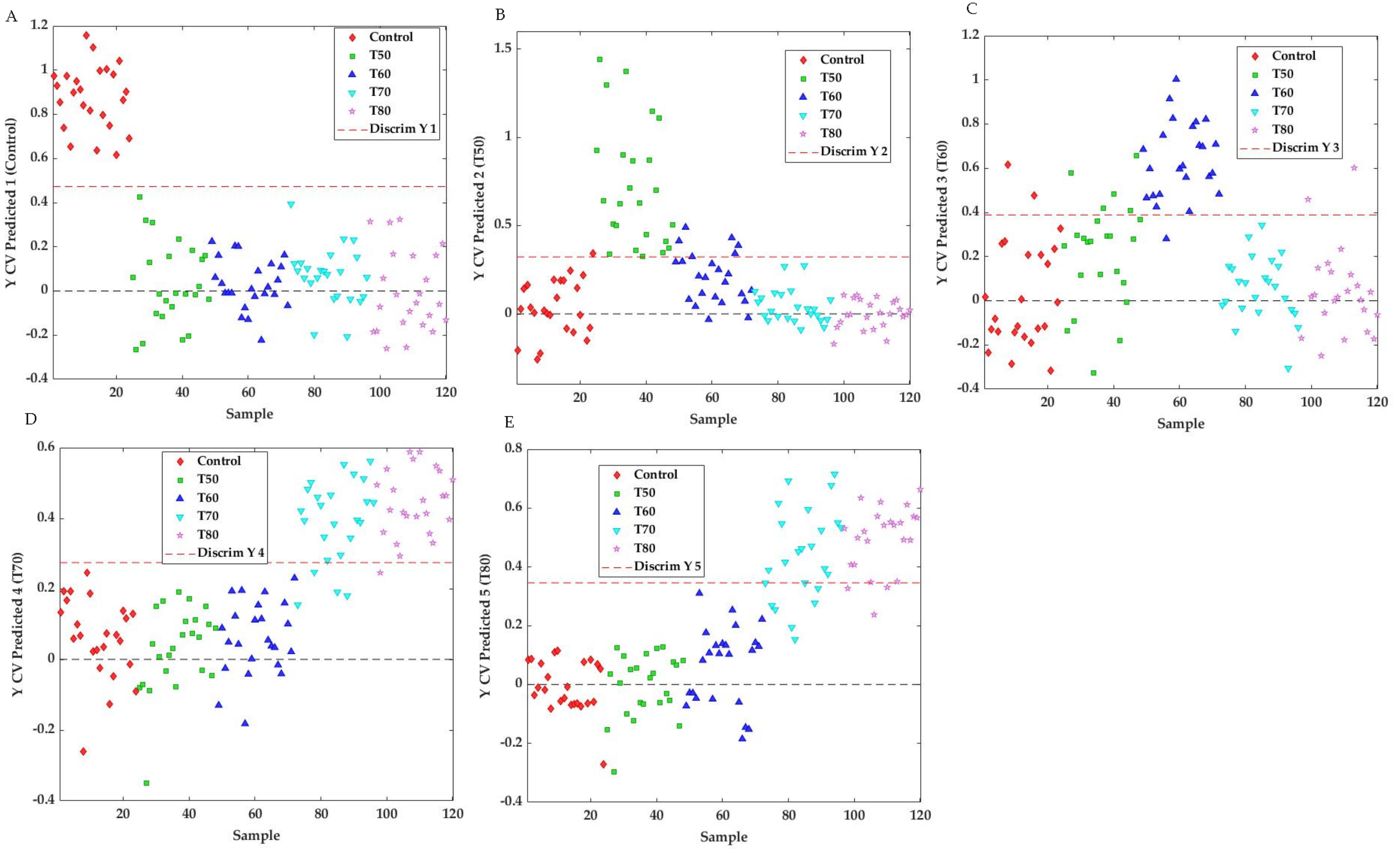

S7: Cross-validated predicted class resulted from application of PLSDA on the diffuse reflectance data, obtained on the different cod groups: A; Control, B; T50, C; T60, D; T70, E; T80.

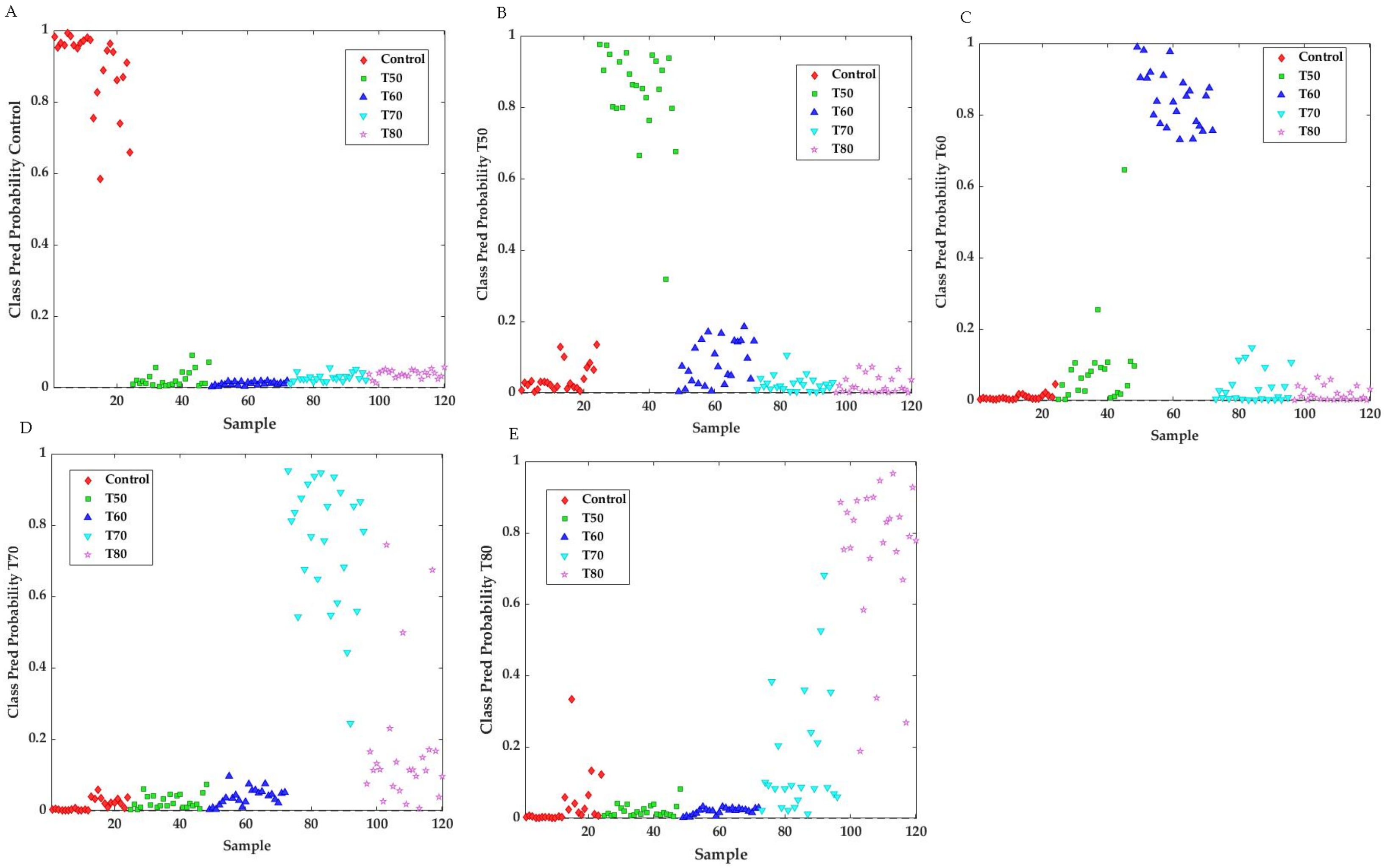

S8: Prediction probability of the SVMC model applied to the fluorescence data, obtained on the different cod groups: A; Control, B; T50, C; T60, D; T70, E; T80.

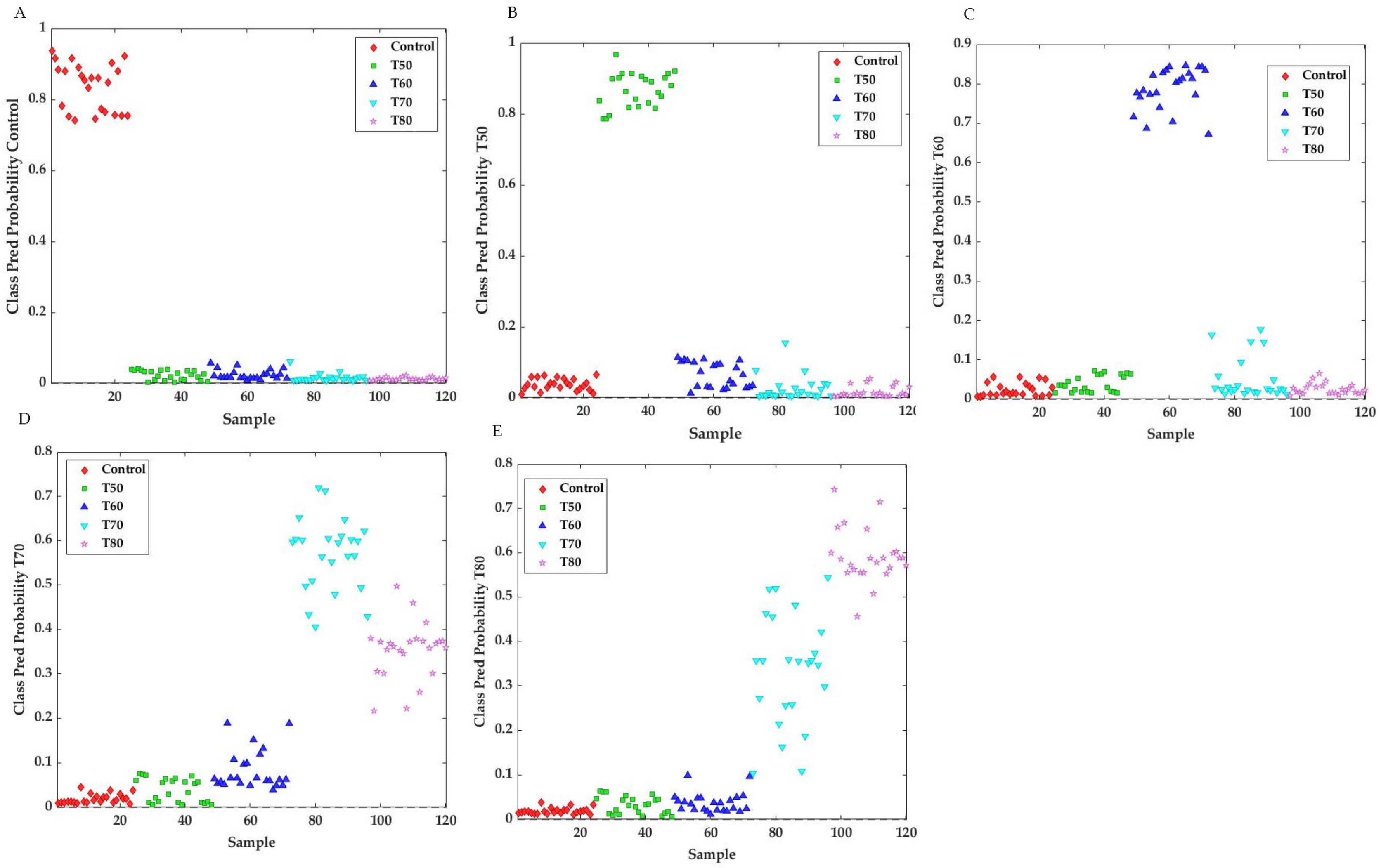

S9: Prediction probability of the SVMC model applied to the diffuse reflectance data, obtained on the different cod groups: A; Control, B; T50, C; T60, D; T70, E; T80.
